# Proteomic and Mutant Analysis of Hydrogenase Maturation Protein Gene *hypE* in Symbiotic Nitrogen Fixation of *Mesorhizobium huakuii*

**DOI:** 10.3390/ijms241612534

**Published:** 2023-08-08

**Authors:** Songhua Long, Min Su, Xiaohong Chen, Aiqi Hu, Fuyan Yu, Qian Zou, Guojun Cheng

**Affiliations:** Hubei Provincial Engineering and Technology Research Center for Resources and Utilization of Microbiology, College of Life Sciences, South-Central Minzu University, Wuhan 430074, China

**Keywords:** *Mesorhizobium huakuii*, hydrogenase maturation protein gene (*hypE*), *energy* and *electrons*, symbiotic nitrogen fixation, proteomics analysis

## Abstract

*Hydrogenases catalyze the simple* yet important redox reaction between protons and electrons and H_2_, thus mediating symbiotic interactions. The contribution of *hydrogenase* to this symbiosis and anti-oxidative damage was investigated using the *M. huakuii hypE* (encoding *hydrogenase* maturation protein) mutant. The *hypE* mutant grew a little faster than its parental 7653R and displayed decreased antioxidative capacity under H_2_O_2_-induced oxidative damage. Real-time quantitative PCR showed that *hypE* gene expression is significantly up-regulated in all the detected stages of nodule development. Although the *hypE* mutant can form nodules, the symbiotic ability was severely impaired, which led to an abnormal nodulation phenotype coupled to a 47% reduction in nitrogen fixation capacity. This phenotype was linked to the formation of smaller abnormal nodules containing disintegrating and prematurely senescent bacteroids. Proteomics analysis allowed a total of ninety differentially expressed proteins (fold change > 1.5 or <0.67, *p* < 0.05) to be identified. Of these proteins, 21 are related to stress response and virulence, 21 are involved in transporter activity, and 18 are involved in energy and nitrogen metabolism. Overall, the HypE protein is essential for symbiotic nitrogen fixation, playing independent roles in *supplying energy* and *electrons, in bacterial* detoxification, and in the control of bacteroid differentiation and senescence.

## 1. Introduction

Molecular hydrogen is an environmentally clean fuel that generates no toxic by-products, and the reversible (bi-directional) hydrogenase (H_2_ases) hold great promise for hydrogen uptake and hydrogen production [1]. *Hydrogenases catalyze the simple* yet important redox reaction between protons and electrons and H_2_ (2H^+^ + 2e^−^ ↔ H_2_) [2]. *Hydrogenases are found throughout* prokaryotes, archaea and lower eukaryotes such as cyanobacteria, sulfate-reducing *bacteria,* and anaerobic fungi [3]. *According to the type of catalytically active metal center, hydrogenases are classified into three classes, the [NiFe]-hydrogenases, the [FeFe]-hydrogenases, and the iron sulfur cluster-free [Fe]-hydrogenases* [4]. *[FeFe]-hydrogenases* mainly produce molecular hydrogen, and *[Fe]-hydrogenases* catalyze a specific reaction utilizing H_2_ [5], whereas [NiFe] hydrogenases present *in the periplasmic space of the bacteria are hydrogen*-*uptake* enzymes that play a crucial role in energy-conservation processes [6].

Biological nitrogen fixation is vital to nutrient cycling in the biosphere and involves the reduction of atmospheric N_2_ to ammonia by the bacterial enzyme nitrogenase [7]. In the nitrogen fixation process, nitrogenases catalyze the reduction of N_2_ with the following limiting stoichiometry: N_2_ + 8H^+^ + 8e^−^ = H_2_ + 2NH_3_ [8]; therefore, a large amount of H_2_ is produced as an obligate by-product of nitrogen fixation. This hydrogen production is a major factor limiting the efficiency of symbiotic nitrogen fixation [9]. Some rhizobia induce a hydrogen uptake (Hup) system with a [NiFe] hydrogenase along with nitrogenases to utilize H_2_ to reduce energy losses [10]. The biosynthesis of [NiFe] hydrogenase is a complex process requiring the function of an 18 gene cluster (*hupSLCDEFGHIJK-hypABFCDEX*) [11]. Among the *hypE* gene cluster, the *hypE* gene is confirmed to be involved in the maturation process of hydrogenase, and may also be involved in the transport of Ni [12].

One of the most interesting and least well-understood of these accessory proteins for the hydrogenase production system is the hydrogenase maturation protein HypE. The pleiotropically acting protein HypE serves an essential function in the biosynthesis of the CN ligands of the active-site iron by catalyzing ATP-dependent dehydration of the carbamoyl group to produce a nitrile group [13]. An *E. coli* mutant *hypE* variant established by amino acid replacements in the nucleoside triphosphate binding region showed no intrinsic ATPase activity [14]. HypE has been involved in the synthesis of the CN ligand, and, additionally, cyano groups from thiocyanate have been transferred to the HypC-HypD complex to modify Fe atoms [15,16]. The HypCDE ternary complex formation results in the opening movements of HypE, which causes the HypE C-terminal tail to adopt the outward conformation, which favors cyanide transfer [15]. The *E. coli hypE* mutant has no hydrogenase activity and exhibits impaired in neutral red-mediated iron reduction activity [17].

The hydrogenase systems in *Bradyrhizobium japonicum* and *R. leguminosarum* show highly conserved sequence and gene organization, and are adequately characterized. In both cases, a membrane-bound heterodimeric [NiFe] hydrogenase is in charge of hydrogen uptake [9]. The *R. leguminosarum hyp* gene cluster is necessary for the production of a functional uptake [NiFe] hydrogenase system and is controlled by the nitrogen fixation regulatory protein NifA [18,19]. It has been shown that the *R. leguminosarum hypA* gene is specifically expressed in bacteroids and required for hydrogenase activity and processing [20], the HypB protein from *B. japonicum* is required for the nickel-dependent transcriptional regulation of hydrogenase [21], and HypC and HypD are involved in the synthesis and transfer of the Fe(CN)_2_CO cofactor precursor [22]. However, the function and mechanism of rhizobial HypE in the symbiotic fixation of nitrogen is not well-established. Here, we investigated the hydrogenase gene *hypE* in another rhizobial genus, *Mesorhizobium*, and the roles of *M. huakuii hypE* in free-living bacteria and during N_2_-fixing symbiosis with *Astragalus sinicus* by analyzing the phenotypes of the *hypE* mutant strain. This study was also designed to characterize the proteome profiling of the nodules of *A. sinicus* in an attempt to uncover the molecular mechanisms regulating nodule formation and development. To our knowledge, this work represents the first proteome analysis of the *hypE* gene in symbiotic root nodules reported to date.

## 2. Results

### 2.1. Bioinformation Analysis of the M. huakuii hypE Gene

*M. huakuii MCHK_8345*, encoding the *hydrogenase maturation* protein HypE, is expressed at high levels during symbiosis. The *hypE* gene is predicted to encode a polypeptide of 355 amino acids, with an expected molecular mass of 36.96 kDa and a theoretical pI value of 5.04. *HypE* catalyzes the synthesis of the CN ligands of the active-site iron of [NiFe] hydrogenases using carbamoylphosphate as a substrate [14]. During the nitrogen-fixation process, rhizobia can induce [NiFe] hydrogenases to recycle the hydrogen evolved by nitrogenase [23].

### 2.2. Growth and Antioxidative Activity in M. huakii hypE Mutant in Free Living Condition

**Figure 1:**
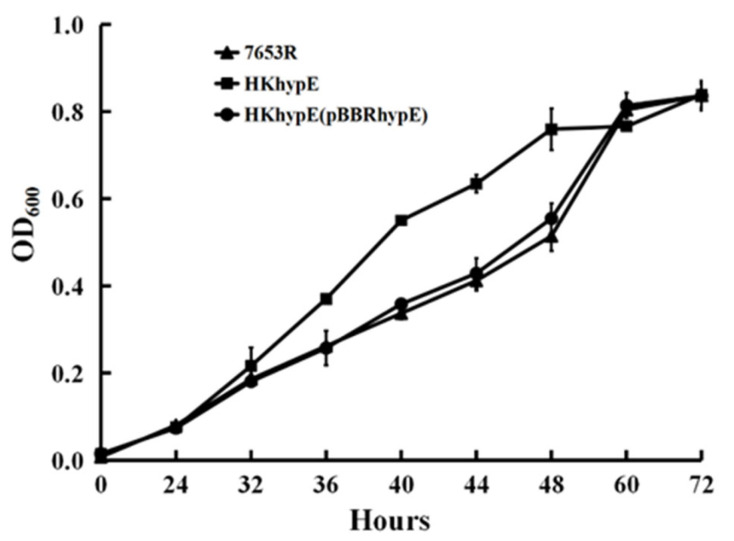
Growth of 7653R, *hypE* mutant HKhypE and complemented strain in AMS medium. Data are from three biological samples plus and minus the standard deviation (±SD).

To experimentally confirm the potential function of the *hydrogenase maturation* protein, a *hypE* gene mutant HKhypE was made *by* means of mutagenesis. The growth of the HKhypE strain was compared with that of wild-type 7653R. In liquid AMS minimal medium with glucose as a carbon source and NH_4_Cl as a nitrogen source, the mutant HKhypE grew slightly faster, and entered a logarithmic growth phase earlier than the parent strain 7653R (Figure 1), while both mutant HKhypE and wild-type strain 7653R achieved the same maximum density. When *hypE* on plasmid (pBBR1MCS-5) was introduced into mutant HKhypE, the resulting strain HKhypE(pBBRhypE) showed the same growth rate as the wild-type 7653R strain (Figure 1).

In order to study the sensitivity to oxidative stress, growth of mutant HKhypE and wild-type strain 7653R was evaluated by disk diffusion method (Table 1). *When H_2_O_2_* was given *at concentrations* of 25, 100, 250, and 1000 mmol/L, the mutant HKhypE showed a clear sensitivity to the H_2_O_2_ treatment, and compared with the wild-type strain 7653R, its growth was significantly inhibited, indicating that HypE has critical roles in protecting cells from hydrogen peroxide stress. *At concentrations* of 25, 100, and 250, the complemented strain HKhypE(pBBRhypE) showed a lower sensitivity compared with the mutant HKhypE (Table 1).

### 2.3. Symbiotic Properties of hypE Mutant Strain

**Figure 2:**
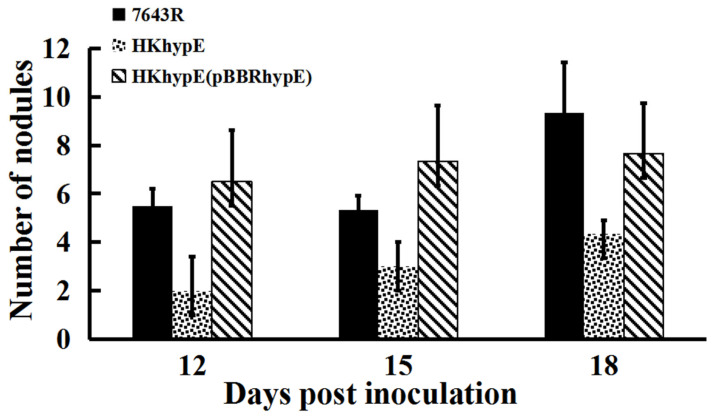
Numbers of nodules per plant, assessed12, 15, and 18 days post inoculation. The data represent means _x0005_ standard deviations (*n* = 32). The experiments were repeated four times, and a representative experiment is shown. Significant differences (*p <* 0.05) were identified by one-way ANOVA, followed by Tukey’s post hoc test, and are noted as different letters (*p <* 0.05).

To determine the function of HypE during symbiotic interactions with an *A. sinicus* host, the symbiotic performance of the *hypE* mutant strain was evaluated. During the early stage of nodule formation (at 12, 15, and 18 days post inoculation), nodule number was significantly reduced via the inoculation of mutant HKhypE compared with that of the wild-type strain (Figure 2). However, when root *nodules* were harvested *28 days* after inoculation, *no* statistically *significant difference was observed* in the number of nodules between plants inoculated with mutant HKhypE and plants inoculated with wild-type 7653R (Table 2). The wild-type strain showed a normal spherical shape of the nodules (2–4 mm), while the *hypE* mutant elicited small-size nodules (0.5–1.5 mm), and the nodule weight per plant inoculated with HKhypE was lower than that inoculated with the wild-type strain. *A. sinicus plants inoculated with* the *hypE mutant* were shorter and thinner, *with* more *yellow leaves*, and the fresh weight of the HKhypE-inoculated plant was 64.39% compared to that of the 7653R-inoculated plant (Table 2). The control plants without rhizobial inoculation had no nodules on their roots and showed clear symptoms of nitrogen deficiency. *A* notable feature of our study was that plants inoculated with mutant HKhypE showed a significant decrease of more than 47% in acetylene reduction activity compared to that inoculated with wild-type 7653R. Plants inoculated with strain HKhypE(pBBRhypE), in which the mutation in *hypE* is complemented by a full-length *hypE* gene cloned on a plasmid, have wild-type properties (Table 2 and Figure 2).

**Figure 3:**
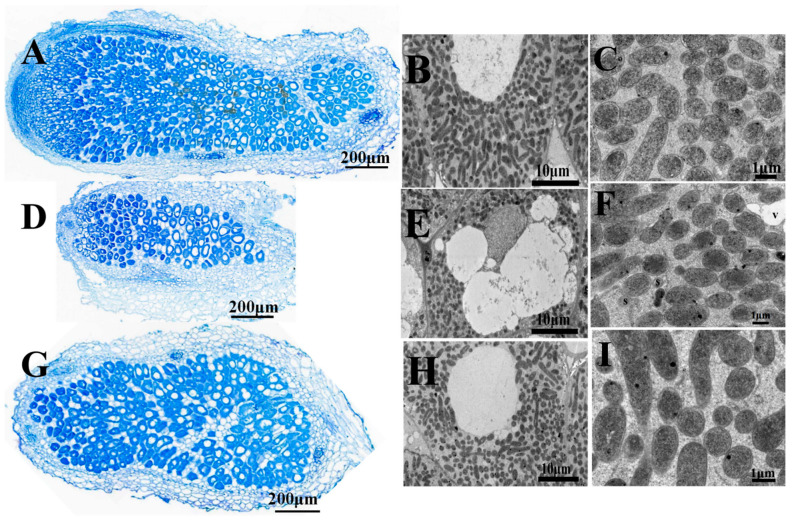
Structure of 4-week-old *Astragalus sinicus* nodules and bacteroids. Nodules were induced by *M. huakuii* 7653R (**A**–**C**), HkhyE (**D**–**F**), HKhypE(pBBRhydA) (**G**–**I**). Scale bars = 200 μm (**A**,**D**,**G**), 10 μm (**B**,**E**,**H**), 10 μm (**C**,**F**,**I**). S, Senescing bacteroid; V, vacuole.

Four-week-old nodules have been examined by light and *scanning* electron microscopy (SEM). Microscopic analysis of the nodules obtained with mutant HKhypE showed that they were small and filled by rhizobia-infected cells, but contained an abnormally thick cortex (Figure 3D). SEM analysis demonstrated nodule cells infected by *hypE* mutant contained several more cavities as compared with nodule cells infected by wild-type 7653R (Figure 3B,E). Moreover, the mutant-infected nodule cells showed signs of early senescence with disintegrating bacteria and vacuolation of infected cells (Figure 3F). The results suggested that the *hypE*-mutant-infected nodules were functionally defective.

### 2.4. Effect of hypE Deletion on H_2_O_2_ Concentration and Glutathione Reductase Activity in Nodules

**Figure 4:**
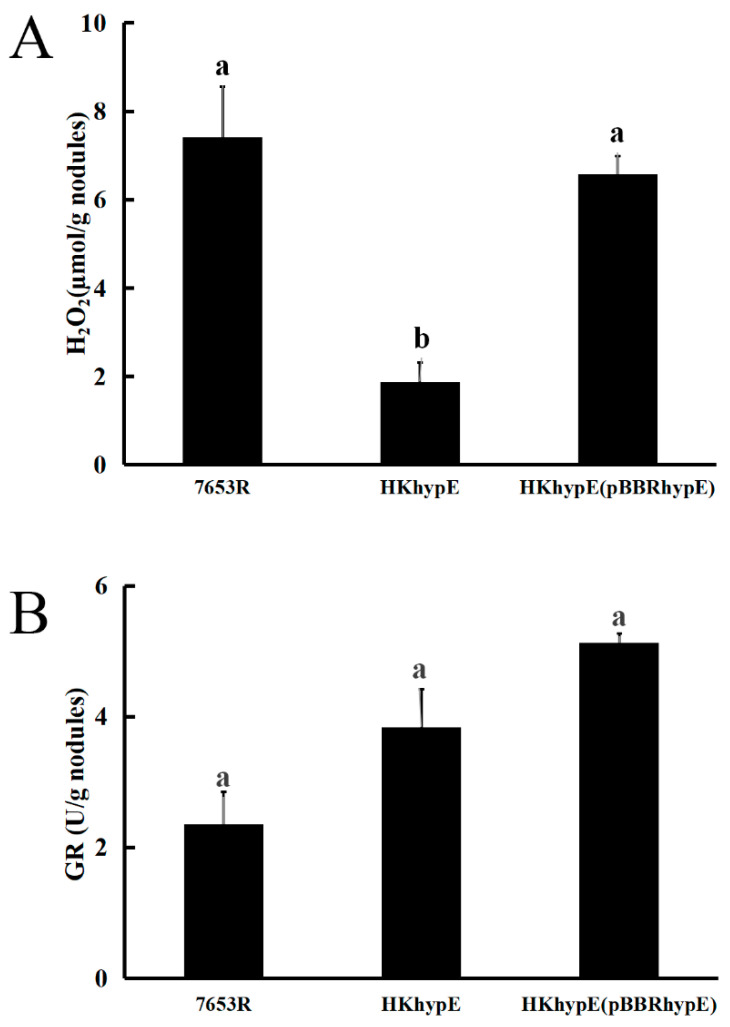
H_2_O_2_ concentration and glutathione reductase activity in *M. huakuii* nodules. (**A**), Levels of H_2_O_2_ in 28-day-old nodules; (**B**), glutathione reductase activity in 28-day-old nodules. Data are the average of three independent biological samples. ^ab^ Different superscript letters indicate significant difference according to ANOVA test (*p* < 0.05).

To investigate the possible reasons for the changes in symbiotic phenotype resulting from *hydrogenase* HypE absence, the H_2_O_2_ content and glutathione reductase activity in the nodules at 28 days post-inoculation were analyzed. The data showed that the glutathione reductase activity had no significant effect in the *hypE* mutant, while the *quantification results of H_2_O_2_* indicated a remarkable decrease in nodules induced by HKhypE compared with plants infected by the wild-type strain (Figure 4). This reduction could be rescued by constitutionally expressing *hypE* from a plasmid in the mutant (Figure 4). These results indicate that HypE is not associated with glutathione reductase activity and that *hydrogenase* maturation protein deficiency abolishes the protective effect of H_2_ against H_2_O_2_-induced oxidative damage.

### 2.5. Rhizosphere Colonization and Competition by M. huakuii Strains

**Figure 5:**
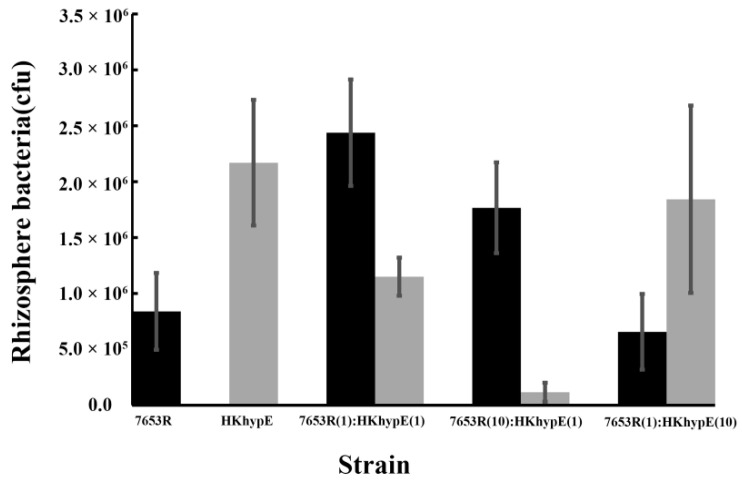
Rhizosphere colonization and competition of the wild-type and the *hypE* mutant HKhypE. The mutant HKhypE and the wild-type for growth in the rhizosphere was measured by inoculating 10^3^ bacteria alone or in mixed strains. Inoculation ratios are given on the *x* axis, with 1 corresponding to 1000 CFU. Seven days after inoculation, the bacterial numbers were measured. Bacterial numbers recovered from 10 plants (mean ± SEM) are shown.

The colonization ability of *M. huakuii* strains for growth and competition in the rhizosphere of *A. sinicus* was evaluated after *inoculating* a low microbial population (10^3^ or 10^4^ bacteria per seedling) into the short-term colonization of *the plant* rhizosphere and counting the total number of bacteria after one week [24]. When the mutant HKhypE and the wild-type 7653R were inoculated alone into the *A. sinicus* rhizosphere, the mutant HKhypE was at a significant advantage (36.37% ration of 7653R to HKhypE) compared to the wild-type 7653R (Figure 5). However, when both *strains were inoculated together* in equal proportion, mutant HKhypE was at a significant disadvantage (53.62% of bacteria recovered) compared to the wild-type 7653R (Student’s *t*-*test*; *p* ≤ 0.01). Even when strain HKhypE was inoculated at a 10-fold excess over 7653R, it accounted for only 273% of bacteria recovered (Figure 5). The results showed that HypE was essential for competition in the host plant *rhizosphere* by *M. huakuii*.

### 2.6. mRNA Expression Levels of HypE Gene in Nodules Induced by M. huakuii 7653R

**Figure 6:**
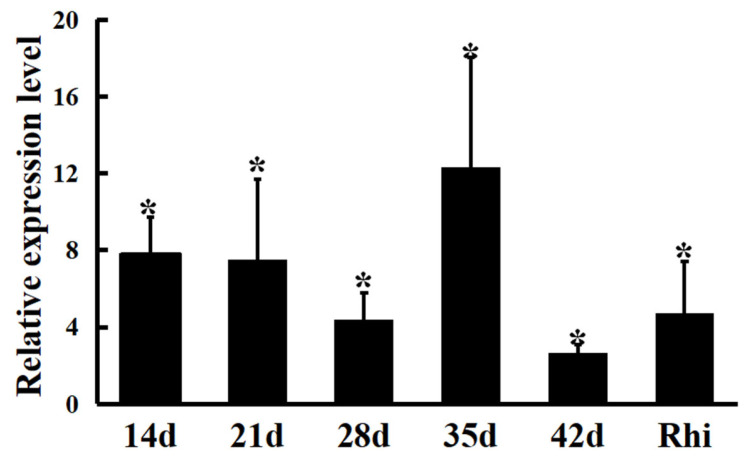
Expression patterns of *hypE* gene in symbiotic nodules and rhizosphere. Gene expression levels were examined via real-time RT-PCR. Nodules were collected on different days after inoculation with 7653R. Relative expression of genes involved in pea rhizosphere or nodule bacteroids at different growth stages compared with 7653R cells growth in AMS Glc/NH_4_^+^. Rhi, rhizosphere strains at 7 days post-inoculation. Data are the average of three independent biological samples (each with three technical replicates). The *gyrB* gene was used for calibration, and * indicates significant difference according to ANOVA test (*p* < 0.05).

The expression levels of the *hypE* gene in wild-type nodules among different growth stages of host plants (14, 21, 28, 35, and 42 days after inoculation) were determined via quantitative real-time fluorescence polymerase chain reaction (qRT-PCR). In all the treatments, the mRNA levels of *hypE* gene were significantly up-regulated by 3.5–12.1 fold as compared to wild-type strains in AMS medium, and the *hypE* gene had the highest expression level (more than 12-fold) in nodules at the nodule maturation stage (35 d) (Figure 6). In the 7-day plant rhizosphere, *hypE* mRNA levels were also increased (4.2 times the control levels, *p* < 0.01) (Figure 6). Therefore, *hypE* gene expression was induced during *A. sinicus-M. huakuii* symbiosis and was indispensable for nodule/bacteroid development and maturation.

### 2.7. Proteomic Analysis of Differential Protein Expression in Nodule Bacteroids

A quantitative proteomic approach *was performed to* examine the influence of HypE deficiency on root *nodule symbiosis.* The high-throughput analytical method was developed using ultra-performance liquid chromatography coupled with tandem mass spectrometry (UPLC-MS/MS) for protein quantification in the HKhypE mutant and 7653R bacteroids. Proteomics analysis allowed the identification of peptides derived from a total of 2852 protein groups in the *hypE* gene mutant and wild-type bacteroids, with molecular weights ranging from 6.3 to 317.0 kDa. A total of ninety differentially expressed proteins (fold change > 1.5 or <0.67, *p* < 0.05) were identified (Table 3). Seven proteins were up-regulated and eighty-three proteins were down-regulated in *hypE* mutant bacteroids. Eighty-two (91.11%) differential *protein*-*encoding genes* were located on the chromosome, and eight (8.89%) *were* localized in symbiotic megaplasmid pMHb. However, no differential *genes were* localized on the megaplasmid pMHa.

To categorize these differences into modules of biological relevance, the 90 differential proteins were assigned to six functional categories, which were mainly involved in stress response and virulence (*n* = 21, 23.33%), energy and nitrogen metabolism (*n* = 18, 20.20%), transporter activity (*n* = 15, 16.67%), carbohydrate metabolism (*n* = 9, 10.00%), nucleotide metabolism (*n* = 8, 8.89%), and unknown function proteins (*n* = 19, 21.11%). In particular, all the differential proteins linked to stress response and virulence are down-regulated in the mutant bacteroids. The number of affected oxidoreductase, hydroperoxide reductase, oxygenase, dehydrogenase, thioredoxin, glutathione S-transferase, and antibiotic biosynthesis monooxygenase also suggests that HypE functions in an antioxidant capacity in the root nodules and that the loss of these proteins could result in antioxidant defect. Further analysis of the differentially expressed genes identified a subset involved in electron transport and nitrogen metabolism. Two genes, *MCHK_2003* and *MCHK_5582*, encoding for nitrogen utilization are up-regulated, while all the proteins relative to electron transport *and nitrogen fixation* ammonia assimilation are down-regulated in the mutant bacteroids. *The nitrogenase* enzyme is composed *of the* Fe and MoFe proteins [25]. Three key nitrogen-fixation genes, *nifX*, *nifD*, and *nifK*, are required for nitrogenase component proteins, and *nifE* is required for the synthesis of the iron–molybdenum cofactor (FeMoco) of nitrogenase. Iron is required for the synthesis of iron-containing proteins in bacteroids for nitrogenase and cytochromes of the electron transport chain [26]. Proteins *MCHK_8220* and *MCHK_4952* associated with iron metabolism were found to show decreased expression in mutant nodules. The number of differentially expressed nitrogenase genes and nitrogen-fixation-associated genes indicated that hydrogenase maturation protein HypE affects the expression of a wide range of genes involved in the legume–*Rhizobium* symbiosis interaction.

Furthermore, 15 of the differentially expressed proteins identified are transport proteins, of which 5 are ABC-type nitrate/nitrite transporters. In addition, MgtE is the lone up-regulated transport protein and has been suggested to be essential for N_2_ fixation [27]. Finally, all the differential expression proteins in the process of nucleotide metabolism were down-regulated in the mutant bacteroids. qRT-PCR was further performed to confirm the validity of the proteome changes. The expression of four genes in four different functional categories were significantly lower in 28-day-old nodules infected by mutant HKhypE compared to wild-type 7653R (Table 3). These results are largely consistent with the changes seen in the proteomic assay results.

## 3. Discussion

In the process of symbiosis with rhizobium, nitrogen fixation is dependent on a source of ATP and the generation of a reductant at low enough redox potential to transfer electrons to nitrogenase [28]. The nitrogenase complex catalyzes the following reactions: N_2_ + 8e^−^ + 16ATP + 16H_2_O → 2NH_3_ + H_2_ + 16ADP + 16Pi + 8H^+^. ATP is required for biological nitrogen fixation processes [29], and large amounts of H_2_ are produced as an obligate by-product of nitrogen fixation in the nodules of legume plants during the nitrogen fixation process. Therefore, this hydrogen production is an important factor limiting the efficiency of symbiotic N_2_-fixation. An important issue is that a [NiFe] hydrogenase HypE, along with nitrogenase, can utilize H_2_ to reduce energy losses, and *hypE* expression is elevated in nitrogen-fixing bacteroids of the root nodules, but the function of HypE in bacteroid nitrogen-fixing systems is poorly understood. Here, we examine the hydrogenase HypE, which is essential for symbiotic nitrogen fixation. Our data demonstrated that HypE is required for root nodule formation and cellular detoxification with regard to its nitrogen fixation capacity and electron transfer.

In this study, mutant HKhypE was constructed using homologous recombination technology, and bioinformatics analysis showed that the HypE protein was involved in the biosynthesis of Ni-Fe hydrogenase and was considered to bind to ATP. The mutation of *M. huakuii hypE* had less influence on the growth of free-living bacteria. Three hydrogenase minus (Hup-) mutants of *Azotobacter chroococcum* also gave similar yields to the parent under N_2_-fixing conditions; however, in carbon-limited mixed cultures, the parent strain outgrew the mutant at high D values, and a *Rhodobacter sphaeroides* Hup~-/Phb~- mutant strain did not grow well and degraded only 19% of acetic acid, implying that hydrogenase has little effect on the steady-state growth but otherwise can be crucially important to the maintenance of a sustainable rate of growth under stress [30,31]. The mutation of *M. huakuii hypE* led to decreased antioxidative capacity under hydrogen peroxide H_2_O_2_ stress. The direct link between hydrogenase and H_2_O_2_ detoxification has been less reported, while in *Oligotropha carboxidovorans*, the reduction of protons to H_2_ by CO dehydrogenase is interpreted as a detoxification reaction for electrons to prevent cell damage [32]. It has been reported that the [FeFe] hydrogenase enzymes are excellent catalysts for H_2_ evolution but rapidly become inactivated in the presence of O_2_ [33].

HypE plays a prominent role in nodulation and nitrogen fixation, as *A. sinicus* plants inoculated with mutant HKhypE exhibited large decreases in the nodule number and nitrogen-fixing activity of rhizobial inoculated plants. The *hypE*-derived mutants formed smaller nodules filled with disintegrating bacteria and vacuolation of infected cells with signs of early senescence. It has been reported that during nitrogen fixation, nitrogenases catalyze the reduction of N_2_ into NH_3_ by using protons and electrons with the evolution of H_2_ [34], [NiFe] hydrogenases, are hydrogen-uptake enzymes that are probably acting to regulate the flow of electrons through electron transport and play a crucial role in energy-conservation processes [35]. Glutathione reductase activity in mutant-inoculated nodules was not different from that wild-type nodules, but the absence of HypE was associated with a 73.7% decrease in nodule H_2_O_2_ content. H_2_O_2_ appears to play an important signaling role in the establishment and the functioning of the interaction between rhizobia and host plants [36]. It has been reported that *A. chroococcurn* hydrogenase can benefit bacteria under N_2_-fixing but not NH_4_^+^-utilizing conditions, suggesting that hydrogenase assists the organism either by providing extra energy or by protecting nitrogenase against the inhibition by O_2_, rather than by protecting nitrogenase against the inhibition of N_2_ reduction by H_2_ [31].

Proteomic experiments were performed to provide a foundation for evaluating the effect of hydrogenase on symbiotic nitrogen fixation. Among the 90 differentially expressed proteins, the “stress response and toxicity”-related proteins in bacteroids induced by mutant HKhypE were down-regulated, and most of the proteins related to “electron transport and nitrogen metabolism”, “transport activity”, “carbohydrate metabolism”, and “nucleotide metabolism” were significantly down-regulated. Firstly, hydrogenase was required to supply energy and electrons for the nitrogen fixation reaction. *phaR* gene was significantly down-regulated, and its expressed protein product regulated the production of polyhydroxyalkanoate (PHA). Studies have shown that *Xanthomonas oryzae* pv. *Oryzae* knocks out the *phaR* gene, showing a decrease in growth rate and a significant decrease in the yield of extracellular polysaccharide [37]. Acidic extracellular polysaccharide is essential for the establishment of nitrogen-fixation symbiosis in leguminous plants. The lack of acidic extracellular polysaccharide will lead to the inability of the *Rhizobium* to effectively recognize specific hosts [38]. The expression products of the *dctA* gene are mainly closely related to the transport of C_4_-dicarboxylate, which is a prerequisite for achieving symbiotic nitrogen fixation [39]. The significant down-regulation of the *dctA* gene is bound to affect the nitrogen-fixation network. Active nitrogen fixation requires a continuous supply of energy and electrons. PHA can be used as a carbon source and energy to provide energy for the nitrogen-fixation network. The significant down-regulation of the *phaR* gene was consistent with the results of electron microscope section experiment in which the bacteroids of HKhypE-mutant-infected nodules were significantly smaller than those of the control group. In addition, C_4_-dicarboxylate plays an isomorphic role in supporting the tricarboxylic acid cycle. Based on the above, it is speculated that the *phaP* and *dctA* genes may affect the efficiency of the nitrogen-fixation network through energy efficiency.

Secondly, the hydrogenase HypE is essential for the development of nodule bacteroids. ExbB forms pentamers as a scaffold to form a Ton system with ExbD and TonB, which is used to transport nutrients such as iron and vitamin B12 [40]. In differential proteomics analysis, the significant down-regulation of the *exbB* gene will undoubtedly hinder the iron transport. In addition, it is interesting to find that the *sbmA* gene is also significantly down-regulated. The SbmA protein is extremely homologous with BacA protein. The *bacA* gene has been proven to be closely related to the early development of bacteroids. A lack of the *bacA* gene will lead to premature senescence of bacteroids in the root nodules [41]. In addition, the *hfq* gene was also found to be significantly down-regulated. Fhq has been reported to be involved in nodule development, the intracellular activity of bacteroids, and nitrogen fixation [42].

Thirdly, HypE plays a prominent role in rhizobial bacteroid detoxification. Thioredoxins act as antioxidants and function as redox regulators in the bacteroids, and the significant down-regulation of the *trxA gene* will cause serious damage to biological macromolecules. The *Phaseolus vulgaris* (common bean) *Trxh* gene family had the highest expression in the nodule primordium (NP), and their expression patterns in the NP were positively correlated with the symbiotic N_2_-fixing efficiency of the *Rhizobium* strain, concomitantly with increased amounts of H_2_O_2_ [43]. Another interesting observation is that the *recR* gene is significantly down-regulated in proteomic analysis. The *recR* gene is involved in regulating the large amount of DNA synthesis and is an indispensable component [44,45]. Among the “transport activity”-related genes, the *mgtE* gene is the only up-regulated transporter, which is predicted to be an *R. leguminosarum* channel and is essential for growth and N_2_-fixation when both Mg^2^⁺ is limited and the pH is low [27].

The *hypE* gene expression is significantly up-regulated during the whole nodulation process, and its highest expression level occurred at 35 days after inoculation. Moreover, the *M. huakuii hypE* mutant was unable to compete efficiently in the rhizosphere with its parent, which shows that rhizobial HypE is essential for the adaptation of the plant host microenvironment. Taken together, *M. huakuii* [NiFe] hydrogenase plays an important role in root nodule symbiosis by providing energy and electrons, ROS and pH-dependent detoxification, and control of bacteroid differentiation and senescence.

## 4. Materials and Methods

### 4.1. Strains, Plasmids, Primers, and Culture Conditions

All the bacterial strains, plasmids, and primers used in this work and their relevant characteristics are listed in Table 4. *M. huakuii* strains were grown at 28 °C in either tryptone yeast extract (TY) [46] or acid minimal salts (AMS) medium [47] supplemented with D-glucose (10 mM) as a carbon source and NH_4_Cl (10 mM) as a nitrogen source. Antibiotics were used at the following concentrations (μg/mL): streptomycin (Str), 250; kanamycin (Km), 20; gentamicin (Gm), 20; neomycin(Neo), 80 or 250 (for generating the *hypE* mutant); spectinomycin (Spe), 100. To monitor culture growth, strains were grown at 28 °C in AMS liquid medium with shaking (200 rpm), and optical density at 600 nm (OD_600_) was measured in three independent cultures.

### 4.2. Construction and Complementation of a hypE Mutant of M. huakuii 7653R

A 657bp *hypE* (*MCHK_8345*) fragment was PCR-amplified using primers hypEfor and hypErev. The fragment was cloned into the *BamH* I and *Hind* III sites of pK19mob, resulting in plasmid pKhypE. Plasmid pKhypE was conjugated into strain 7653R via triparental mating using the helper plasmid pRK2013, as previously described [47]. Insertions into *hypE* gene of strain 7653R were confirmed via PCR using hypEmap and a pK19mob-specific primer (either pK19A or pK19B).

To construct a plasmid for complementation of the *hypE* mutant, a 1.82 kb DNA fragment of the complete *hypE* gene was PCR-amplified from the genomic DNA of *M. huakuiii* 7653R using primers chypEfor and chypErev. The PCR product was cloned into *Xba* I and *BamH* I sites of pBBR1MCS-5, and the resultant plasmid was named pBBRhypE. Plasmid pBBRhypE was conjugated into the HKhypE recipient strain via triparental mating using pRK2013 as a helper plasmid. Using selection for gentamicin resistance, complemented strain HKhypE(pBBRhypE) was isolated as previously described [52].

### 4.3. Cellular Sensitivity to H_2_O_2_

The H_2_O_2_ resistance assay was performed using the disk diffusion method as previously described by *M. huakuiii* 7653R, and HKhypE and HKhypE(pBBRhypE) were grown aerobically in AMS Glc/NH_4_^+^ [53]. An amount of 100 µL of each bacterial suspension (approximately OD_600_ = 0.4) was spotted onto solid TY medium. Sterile *paper disks* of *6 mm in diameter were* laid on the inoculated plates. A total of 10 µL of 25, 100, 250, 1000 mM H_2_O_2_ was *pipetted onto* the surface of separate disks. *The plates were incubated at* 28 °C until circular clear zones could *be* observed. *The diameter of zone of inhibition* (mm) observed was measured for the mutant and compared with that for wild-type 7653R to provide an estimate of its relative susceptibility to oxidants. The experiment was repeated three times, and the data were analyzed using two-way ANOVA (*p* < 0.05).

### 4.4. Plant Experiments and Microscope Study of Nodules

Seeds of *Astragalus sinicus* were surface-sterilized for 5 min in 75% ethanol, soaked 20 min in 2% sodium hypochlorite, and then rinsed 10 times with sterile water. The plants were grown in 500 mL pots containing sterile vermiculite and watered with nitrogen-free Fahraeus solution. Inoculation with *M. huakuii* strains was conducted on 7-day-old seeds. Plants were incubated in a controlled-environment chamber with a cycle of 8 h at 20 °C in the dark and a 16 h photoperiod at 22 °C in the light. *Number of* root *nodules in the early* growing *stage* was *counted at* 12, 15, and 18 days post-inoculation. At 28 days post-inoculation, *nodule* number *per plant*, nodule *fresh weight per plant,* f*resh weight per plant*, and above-ground *fresh weight per plant* were *measured.* Acetylene reduction activity was determined by gas chromatographic measurement, as previously described [54]. Briefly, The plants were extracted and placed in a 50 mL milled bottle, and 1.28 mL of acetylene was injected into the bottle, which was then incubated in a growth chamber at 28 °C for 1 h. Then, 1 mL of gas from the bottle was aspirated and injected into a SP-2100A gas chromatograph (Beijing Beifen-Ruili Analytical Instrument (Group) Co., Ltd., Beijing, China), and the ethylene peak area was separated and detected using an OV-101 capillary column (1.5 m × 0.32 mm × 0.5 μm). The experiment consisted of two independent experiments, each of which had at least five repeats, and *statistical differences were* analyzed using *two-way ANOVA* (*p*  <  *0.05*).

Root *nodules* at 28 days post-inoculation were obtained and examined by the use of both light and electron microscopes. *Nodules were washed and fixed in 2.5% glutaraldehyde, postfixed in 1.5% osmium tetroxide, and* frozen under liquid nitrogen. Semi-thin sections (1–3 µm) of nodules were cut, stained with toluidine-blue solution, and evaluated using a light microscope (SZX16, Olympus Corporation; Tokyo, Japan). *Ultra-thin sections were stained with* uranyl acetate and *lead citrate and* examined using *a Hitachi H-7100 transmission electron microscope.*

### 4.5. Measurement of H_2_O_2_ Concentration and Glutathione Reductase Activity in the Nodules

The roots of *7-day-old seedlings were inoculated* with *M. huakuii* strains. *Nodules* at 28 *days post-inoculation were* collected, *ground* into fine powders in liquid nitrogen, and then *suspended in* precooled extraction buffer (10 *mM Tris-HCl, pH 7.0*). H_2_O_2_ concentration and glutathione reductase activity were measured using corresponding kits (catalogue numbers BC3590-50 for *H_2_O_2_ Content* Assay and BC1160-50 for glutathione reductase activity, Solarbio life sciences) following the manufacturers’ instructions. The experiment was repeated three times, and the data were analyzed using two-way ANOVA (*p*  <  0.05).

### 4.6. Rhizosphere Colonization

Rhizosphere colonization was *performed as* described previously [53]. *A. sinicus* seedlings were germinated and grown in 20 mL centrifuge tubes filled with sterile vermiculite, as described above for acetylene reduction. The 7 day-old plants were inoculated with *M. huakuii* 7653R and HKhypE in the cfu ratios 1000:0, 0:1000, 1000:1000, and 1000:10,000. After 7 days (14 days post-inoculation), shoots were cut off, and 10 mL of sterile phosphate-buffered saline (PBS) buffer (pH 7.4) was added to the roots and vortexed for 15 min [55]. After vortexing, the samples were serially diluted and plated on TY medium containing either streptomycin or streptomycin and neomycin, giving the total number of viable rhizosphere- and root-associated bacteria. The plates were *incubated* at 28 °C for 72 h before *the* colonies were *counted*. *Each treatment consisted of* ten replications, and *each test* consisted of a single plant. *Statistical differences were* evaluated using *one-way ANOVA* (*p  *<  *0.05*).

### 4.7. RNA Isolation and Quantitative Reverse Transcription-PCR (q*RT-PCR*)

*qRT-PCR* was used *to determine* differences in the expression of genes. *M. huakuii *samples* were collected* in triplicates from free-living *M. huakuii* 7653R cultured in AMS medium, *rhizosphere strains at 7 days post-inoculation, and plant nodules,* which were harvested from *A. sinicus* inoculated with *M. huakuii* after 14, 21, 28, 35, and 42 days. The nodules of plants were *ground* into a fine powder with *liquid nitrogen.* Total RNA was isolated using the Trizol Reagent (Invitrogen). First-strand cDNA synthesis and double-strand cDNA amplification were performed using the PrimeScript RT reagent kit with gDNA Eraser (Takara, Dalian, China) according to the manufacturer’s instructions. qRT-PCR was performed using *SYBR Premix ExTaq* kit (Takara, Dalian, China) following the manufacturer’s instructions on the BIO-RAD CFX96 Real-Time PCR Detection System. The sequences of primers for qRT-PCR were listed in Table 4. The *gyrB* gene of *M. huakuii* was used as a reference. For each experiment, three independent biological replicates were performed, and the relative expression levels *of the* mRNAs of the target genes were normalized using the 2^−ΔΔCT^ method.

### 4.8. Protein Extraction, Digestion, and Peptide Labeling

The 28-day nodules *induced by wild-type 7653R or mutant HKhypE* were harvested and ground into fine powder in liquid nitrogen. The tissue powder was transferred into a 5 mL ice-cold centrifuge tube. Four volumes of lysis buffer (1% protease inhibitor cocktail in 8 M urea) were then added to the cell powder, and the lysates were sonicated three times on ice using a high-intensity ultrasonic processor. The insoluble fraction was removed via centrifugation in a cooled centrifuge (at 12,000 rpm for 10 min at 4 °C). Then, the clarified supernatant was collected, and the total protein content was quantified using a BCA protein assay kit (Pierce, Rockland, IL, USA). Equal amounts of proteins were reduced with 10 mM dithiothreitol (DTT) and alkylated with 11 mM iodoacetamide (IAA) for 15 min at room temperature in the dark. *The protein sample was diluted* with 100 mM *tetraethyl ammonium bromide (TEAB*) so that the final concentration of urea was less than 2 M. For digestion of protein, trypsin was added at a protein-to-enzyme ratio of 100:1 (100 μg total protein *was added to* 1 μg *trypsin*), and the digestion was performed overnight at 37 °C. After digestion, the peptides were desalted using a Strata X C18 SPE column *and vacuum-dried*. Then, the dried peptide was reconstituted in 0.5 M TEAB and processed by following the manufacturer’s protocol of the TMT kit (ThermoFisher Scientific, Bremen, GA, USA). Briefly, one unit of tandem mass tag (TMT) reagent, together with 100 μg of sample peptides, was dissolved and reconstituted in acetonitrile. After incubation at room temperature for 2 h, *the peptide mixtures* were desalted *and* dried via vacuum centrifugation.

### 4.9. Fractionation of Tryptic Peptides and LC-MS/MS Analysis

The labeled peptide was mixed and fractionated via high-performance liquid chromatography (*Thermo Scientific EASY-nLC 1000*) *using an Agilent 300 Extend C18 column* (5 μm × 4.6 mm × 250 mm). Concisely, the peptides were first separated using *a gradient* of 2–60% acetonitrile in ammonium bicarbonate (10 mM, pH 10). *The extracts were* fractionated *into* 80 *fractions* in 80 min, and these *fractions were then* combined into 18 fractions and dried via vacuum centrifugation.

The tryptic peptides were resuspended in buffer *solvent* A (30% acetonitrile, 70% water, 0.1% formic acid) and separated with a reversed-phase analytical column (*75 μm* × *15 cm*). For whole-cell proteome analysis, the gradient was run at a constant flow rate of 400 nL min^−1^ for 45 min, starting from 6% to 22% solvent B (0.1% formic acid in 98% acetonitrile) over 26 min, followed by 23% to 35% solvent B in 8 min, and increased to 80% solvent B in 3 min, then maintained at 80% solvent B for another 3 min.

The eluted peptides were further analyzed using a Q Exactive™ plus tandem mass spectrometer (Thermo Fisher Scientific, Massachusetts, USA) coupled with ultra-performance liquid chromatography (UPLC) (Thermo Scientific EASY-nLC 1000, Massachusetts, USA). UPLC was performed using homemade analytical column with integrated spray tip (100 μm i.d. × 25 cm) packed with 1.9 μm/120 Å ReproSil-PurC18 resins (Dr. Maisch GmbH, Ammerbuch, Germany). The intact peptides were detected in the Orbitrap at a high resolution of 70,000. Peptides were selected for MS/MS using a normalized collision energy (NCE) setting of 28, and ion fragments were detected in the Orbitrap at a low resolution of 17,500. A data-dependent procedure that alternated between one MS scan followed by 20 MS/MS scans with 10 s dynamic exclusion, and the electrospray voltage applied was 2.0 kV. In order to prevent overfilling of the ion trap, automatic gain control (AGC) was set to accumulate 5 × 10^4^ ions for generation of MS/MS spectra. For the full-scan mode, the mass range for the MS scans was 350 to 1800 m/z, and the *MS analysis alternated between MS and data-dependent MS2 scans using dynamic exclusion. For each sample, three independent biological replicates were performed.*

### 4.10. Data Analysis

The resulting MS/MS data were processed *using the MaxQuant* search engine (version 1.5.2.8). Tandem mass spectra were searched against the *M. huakuii* 7653R genome database. Trypsin/P was specified as a cleavage enzyme allowing up to 4 missing cleavages. *The mass error was set to* 20 ppm *for the precursor ions, and* the mass tolerance was set to 0.05 Da *for the* MS/MS *fragment ion* matches. Carbamidomethylation of cysteine was selected as fixed modification, while methionine oxidation was set as variable modification. TMT 6-plex was selected in Mascot for the protein quantification method. A *false discovery rate (FDR)* of 1% was imposed *for protein* and *peptide identifications*. The *minimum peptide length was set to 7*, and *the* minimal *peptide score for modified peptides was set* to 40. *The site localization probability was set to > 0.75.* Statistical significance for protein *expression was analyzed using* Student’s *t* tests (*p* < 0.05). *Only proteins* identified in at least two of three *biological replicates with a fold change* higher than 1.5 or lower than *0.67 were considered to* indicate statistically significant difference *in protein abundance between mutant and wild-type strains.* The mass spectrometry proteomics data have been uploaded to the ProteomeXchange Consortium via the PRIDE partner repository with the dataset identifier PXD026564.

## 5. Conclusions

We discovered that the mutant in the hydrogenase maturation protein HypE can affect symbiotic nitrogen fixation between rhizobia and host via the formation of smaller and defective nodules. The *hypE* mutant displayed decreased antioxidative capacity and competition ability in the host plant rhizosphere. The proteomic results show that HypE is mainly involved in stress response and virulence, energy and nitrogen metabolism, and transporter activity. As a result, we believe that *M. huakuii* [NiFe] hydrogenase HypE plays an important role in root nodule symbiosis by providing energy and electrons, ROS, and control of bacteroid differentiation and senescence.

## Figures and Tables

**Table 1:** The inhibition zone diameters of *M. huakuii* stains in different concentrations of H_2_O_2_.

Strain	Diameter (cm)			
	25	100	250	1000
7653R	0.96 ± 0.09 ^a^	2.12 ± 0.17 ^a^	3.04 ± 0.21 ^a^	4.22 ± 0.17 ^a^
HKhypE	2.13 ± 0.25 ^b^	2.75 ± 0.12 ^b^	3.88 ± 0.18 ^b^	4.73 ± 0.11 ^b^
HKhypE(pBBRhypE)	1.40 ± 0.26 ^a^	2.60 ± 0.28 ^ab^	3.47 ± 0.51 ^ab^	5.30 ± 0.61 ^b^

Data are averages (±SEM) from 3 independent experiments. ^a,b^ Different superscript letters in the same row indicate significant difference (two-way ANOVA, *p* < 0.05).

**Table 2:** Symbiotic phenotype of 7653R and HKhypE ^α^.

Strain *M. huakuii*	*The Aboveground Fresh Weight* per Plant (mg) ^β^	Number of Total Nodules per Plant ^β^	Acetylene Reduction Activity (nmol of Ethylene/Plant/h) ^β^	Nodule Fresh Weight per Plant (mg of Plant) ^β^	Fresh *Weight* (mg of Plant) ^β^
7653R	96.41 ± 23.97 ^a^	17.5 ± 3.5 ^a^	64.87 ± 10.9 ^a^	12.0 3± 1.18 ^a^	129.05 ± 16.55 ^a^
HKhypE	77.56 ± 20.53 ^a^	11.7 ± 3.8 ^a^	34.20 ± 0.72 ^b^	10.10 ± 1.56 ^a^	83.10 ± 20.68 ^ab^
HKhypE(pBBRhypE)	98.70 ± 18.52 ^a^	13.7 ± 2.1 ^a^	52.70 ± 7.50 ^a^	17.53 ± 9.28 ^a^	124.03 ± 21.77 ^a^
Control ^γ^		0	0	0	46.08 ± 13.60 ^b^

^α^ All data are averages (± SEM) from at least ten independent plants. Acetylene reduction activity of nodules induced by *hypE* mutant strain HKhypE was compared to that of nodules induced by the wild-type strain 7653R. ^β a,b^ Values in each column followed by the same letter are not significantly different (*p* ≤ 0.05). ^γ^ Control: without inoculation.

**Table 3:** Differential expression proteins in 4-week nodule *hypE* mutant bacteroids relative to wild-type bacteroids ^α^.

Gene ID	Gene Name	Protein Description	MW [kDa]	Ratio	*p* Value	qRT-PCR
**Stress response and virulence**						
MCHK_6427		Formate dehydrogenase	17.09	0.67	0.000082	
MCHK_0466		Isopenicillin N synthase family oxygenase	37.22	0.67	0.000192	
MCHK_4591		Oxidoreductase	27.82	0.67	0.000901	
MCHK_4254		Accessory factor	10.76	0.67	0.000011	
MCHK_10150		Glutathione S-transferase	24.74	0.66	0.001701	
MCHK_6266	*sfnG*	Dimethyl sulfone monooxygenase	40.68	0.66	0.000086	
MCHK_1994		Thioredoxin domain-containing protein	28.47	0.66	0.000105	
MCHK_4344		Antibiotic biosynthesis monooxygenase	10.83	0.66	0.018067	
MCHK_8201 *		Cold-shock protein	7.46	0.65	0.007124	
MCHK_6140	*trxA*	Thioredoxin	11.45	0.66	0.000015	
MCHK_4761		Copper chaperone PCu(A)C	18.79	0.63	0.000415	
MCHK_1937		Competence/damage-inducible protein	26.35	0.63	0.000727	
MCHK_4145		Cold-shock protein	7.36	0.62	0.000013	
MCHK_6264	*ssuD*	Alkanesulfonate monooxygenase	42.28	0.61	0.000139	
MCHK_3694		Universal stress protein	15.68	0.61	0.001310	
MCHK_1730	*bamE*	Outer membrane protein assembly factor BamE	18.38	0.60	0.000001	
MCHK_3913		Response regulator	22.20	0.58	0.012234	
MCHK_6272		Sulfur acquisition oxidoreductase	44.72	0.58	0.000003	
MCHK_4242		Cold-shock protein	7.36	0.56	0.000046	
MCHK_5101		Alkyl hydroperoxide reductase	24.02	0.55	0.000007	
MCHK_3970		Blue-light-activated histidine kinase	38.90	0.31	0.000327	
**Electron transport and nitrogen metabolism ^β^**						
MCHK_2003		Peptide chain release factor 2	41.93	1.59	0.000011	
MCHK_5582	*asd*	Aspartate-semialdehyde dehydrogenase	37.68	1.56	0.000005	
MCHK_8172 *	*nifE*	Nitrogenase iron-molybdenum cofactor biosynthesis protein	54.25	0.76	0.000009	
MCHK_8175 *	*nifD*	Nitrogenase protein alpha chain	55.52	0.75	0.000001	
MCHK_11255 *	*nifX*	Nitrogen fixation protein NifX	18.30	0.74	0.000258	0.19
MCHK_8174 *	*nifK*	Nitrogenase molybdenum-iron protein beta chain	57.54	0.73	0.000005	
MCHK_1461		Glycine-zipper protein	10.77	0.67	0.007390	
MCHK_4867	*argJ*	Arginine biosynthesis bifunctional protein ArgJ	43.53	0.67	0.000191	
MCHK_1729	*hppA*	K(+)-insensitive pyrophosphate-energized proton pump	72.53	0.67	0.000133	
MCHK_2808		TonB-dependent hemoglobin/transferrin/lactoferrin receptor	78.12	0.63	0.000009	
MCHK_4872		Parvulin-like PPIase	32.62	0.63	0.000006	
MCHK_8220 *		Ferredoxin-like protein	11.22	0.63	0.000046	
MCHK_4952		Iron–sulfur metabolism protein	11.03	0.61	0.000100	
MCHK_4282		Cytochrome b	48.57	0.60	0.000122	
MCHK_5860		Lipoprotein	30.60	0.60	0.000001	
MCHK_2579		NAD(P)H nitroreductase	21.17	0.59	0.004028	
MCHK_5859		ATP-binding cassette protein	39.11	0.57	0.001336	
MCHK_12735 *		Nif11-like leader peptide family natural product	12.54	0.50	0.000007	
**Transporter activity**						
MCHK_2240	*mgtE*	Magnesium transporter	49.34	1.59	0.004613	
MCHK_6148		PTS fructose transporter	14.15	0.66	0.000040	
MCHK_5406	*exbB*	Biopolymer transport protein	26.06	0.65	0.000174	
MCHK_0751	*dctA*	C4-dicarboxylate transport protein	46.13	0.65	0.004547	0.30
MCHK_5366		MFS transporter	48.53	0.64	0.002941	
MCHK_0068		Sugar ABC transporter permease	44.82	0.64	0.000491	
MCHK_0065		Transporter substrate-binding protein	27.37	0.64	0.000205	
MCHK_5842		Extracellular solute-binding protein	45.46	0.60	0.000002	
MCHK_1547		ABC transporter substrate-binding protein	34.13	0.59	0.000174	
MCHK_1725		ABC transporter substrate-binding protein	31.80	0.56	0.000000	
MCHK_4896		Extracellular solute-binding protein	35.20	0.56	0.000000	
MCHK_0900	*sbmA*	Peptide antibiotic transporter	47.59	0.55	0.000059	
MCHK_0625		Transporter substrate-binding domain-containing protein	34.91	0.52	0.000281	
MCHK_5677	*tauA*	Taurine ABC transporter substrate-binding protein	35.37	0.51	0.000039	
MCHK_3276		Sulfate ABC transporter substrate-binding protein	36.02	0.51	0.000002	
**Carbohydrate metabolism**						
MCHK_4339		Anthranilate synthase	81.05	2.32	0.000014	
MCHK_3715		N-acetyltransferase	17.31	1.58	0.018854	
MCHK_6064		Phospho-2-dehydro-3-deoxyheptonate aldolase	38.82	1.53	0.000117	
MCHK_5496	*leuC*	2-methyl-cis-aconitate hydratase	50.89	1.50	0.000007	
MCHK_1778		Enoyl-CoA hydratase	29.06	0.67	0.000008	
MCHK_4672		Tripartite tricarboxylate transporter substrate binding protein	33.48	0.66	0.000291	
MCHK_4592		Dehydratase	17.62	0.65	0.017197	
MCHK_5108		Fructose-bisphosphate aldolase	36.32	0.63	0.000001	
MCHK_5188	*phaR*	Polyhydroxyalkanoate synthesis repressor PhaR	23.01	0.54	0.000042	0.45
**Nucleotide metabolism**						
MCHK_5898		Ribosome biogenesis GTP-binding protein	23.76	0.67	0.002586	
MCHK_3722		Ester cyclase	14.75	0.67	0.014111	
MCHK_5344		Pyridoxal phosphate homeostasis protein	23.62	0.66	0.001619	
MCHK_4655	*cdd*	Cytidine deaminase	13.98	0.66	0.000198	
MCHK_6508	*recR*	Recombination protein RecR	21.46	0.65	0.000427	
MCHK_1792		DNA-binding protein HU	9.18	0.63	0.000009	
MCHK_2180	*hfq*	RNA chaperone Hfq\	11.57	0.63	0.000042	0.40
MCHK_3965		Transcriptional regulator	23.35	0.27	0.000081	
**Unknown function proteins**						
MCHK_2160		Uncharacterized protein	13.09	0.67	0.001235	
MCHK_3617		Uncharacterized protein	7.02	0.67	0.003551	
MCHK_1009		Uncharacterized protein	14.09	0.67	0.000005	
MCHK_3463		Uncharacterized protein	11.22	0.66	0.000455	
MCHK_5458		Uncharacterized protein	10.60	0.64	0.000965	
MCHK_3109		Uncharacterized protein	11.20	0.64	0.000074	
MCHK_6262		Uncharacterized protein	14.25	0.64	0.000203	
MCHK_1978		Uncharacterized protein	15.74	0.64	0.000033	
MCHK_4545		Uncharacterized protein	17.29	0.64	0.000246	
MCHK_6162		Uncharacterized protein	7.90	0.64	0.000537	
MCHK_0805		Uncharacterized protein	37.47	0.63	0.000066	
MCHK_1574		Uncharacterized protein	24.45	0.63	0.000404	
MCHK_5383		Uncharacterized protein	23.59	0.62	0.000388	
MCHK_6128		Uncharacterized protein	18.16	0.61	0.000374	
MCHK_5147		Uncharacterized protein	6.78	0.61	0.000204	
MCHK_1260		Uncharacterized protein	7.60	0.60	0.000010	
MCHK_5627		Uncharacterized protein	18.69	0.59	0.001819	
MCHK_5063		Uncharacterized protein	11.40	0.57	0.000147	
MCHK_12690 *		Uncharacterized protein	9.32	0.47	0.000240	

^α^ Protein *expression* was analyzed statistically *using* Student’s *t* tests (*p* < 0.05). All proteins except four NifEDFK with a fold change > 1.5 or <0.67 were considered significantly differentially expressed. ^β^ Four proteins NifEDFK with a fold change < 0.77 were considered down-regulated. * Gene is located in symbiotic megaplasmid pMHb. The bold font in the table represents the functional classification of proteins.

**Table 4:** Strains, plasmids, and primers used in this experiment.

Strains	Description	Reference, Source, Sequence
*M. huakuii* 7653R	Wild type, Nod^+^ on *Astragalus sinicus*	[48]
*M. huakuii* HKhypE	7653R *hypE*:pk19mob, Str^r^ Neo^r^	This study
*M. huakuii* HKhypE(pBBRhypE)	HKhypE carrying pBBRhypE; Str^r^ Neo^r^ Gm^r^	This study
DH5α	F^−^ *lacZ*DM15 *recA1 hsdR17 supE44* D(*lacZYA argF*)	This study
Plasmids		
pK19mob	pUC19 derivative *lacZ mob* Km^r^	[49]
pRK2013	Helper plasmid for mobilizing plasmids Km^r^	[50]
pKhypE	hypEfor/hypErev PCR product in pK19mob, Km^r^	This study
pBBR1MCS-5	*lacPOZ*′ *mob, broad host range*, Gm^r^	[51]
pBBRhypE		
Primer *		
hypEfor	Sense primer for *hypE* mutation	TTTAAGCTTATCGAGGAAGGCATGAAGG
hypErev	Antisense primer for *hypE* mutation	TTTTCTAGACTGCATGGTCACGCGCCCCG
hypEmap	Mapping PCR primer for *hypE* mutation	GCCAAGCCGCTCTATCTGTC
pK19A	pK19mob mapping primer	ATCAGATCTTGATCCCCTGC
pK19B	pK19mob mapping primer	GCACGAGGGAGCTTCCAGGG
chypEfor	Sense PCR primer for complementation of *hypE* mutant	TTTGGATCCGGTGATCATGGTCATGCGAA
chypErev	Antisense PCR primer for complementation of *hypE* mutant	TTTTCTAGACAGTATGGCGGCGTCAAGAA
M13-F	Sense primer for *LacZ*	CGCCAGGGTTTTCCCAGTCACGAC
M13-R	Antisense primer for *LacZ*	CACACAGGAAACAGCTATGAC
QhypE_F	Sense primer for qRT-PCR of *hypE*	TGAAAGACCTGATCGACGAC
QhypE_R	Antisense primer for qRT-PCR of *hypE*	CAAGCCGGTCGCCATGTTTT
QgyrB_F	Sense primer for qRT-PCR of *gyrB*	TTCGACCAGAATTCCTACAA
QgyrB_R	Antisense primer for qRT-PCR of *gyrB*	GCTCATTTCGAAGATCTGGC
MCHK_11255F	Sense primer for qRT-PCR of *MCHK_11255*	GCCTCTCACTCGTCACTGAC
MCHK_11255R	Antisense primer for qRT-PCR of *MCHK_11255*	GCCGAAATGGGCATTGAGGT
MCHK_0751F	Sense primer for qRT-PCR of *MCHK_0751*	AAGATGATCATCGCCCCGGT
MCHK_0751R	Antisense primer for qRT-PCR of *MCHK_0751*	CCAGCGTCGAGAAGGTGAGG
MCHK_5188F	Sense primer for qRT-PCR of *MCHK_5188*	GGGGACGAGCACCTATGTGA
MCHK_5188R	Antisense primer for qRT-PCR of *MCHK_5188*	AAAATGATCTGAGTCAGCAC
MCHK_2180F	Sense primer for qRT-PCR of *MCHK_2180*	GATGATGTTTTCCCAGGTCA
MCHK_2180R	Antisense primer for qRT-PCR of *MCHK_2180*	TGCCCATCAACGTGCATGTG

* Restriction sites in primer sequences are underlined.

## Data Availability

The datasets presented in this study can be found in online repositories. The names of the repository/repositories and accession number(s) can be found below: https://www.ebi.ac.uk/pride/archive/projects/PXD026564 (accessed on 8 August 2021).
